# Protein Composition of Circulating Extracellular Vesicles Immediately Changed by Particular Short Time of High-Intensity Interval Training Exercise

**DOI:** 10.3389/fphys.2021.693007

**Published:** 2021-07-01

**Authors:** Yoshinao Kobayashi, Akiko Eguchi, Yasuyuki Tamai, Sanae Fukuda, Mina Tempaku, Kiyora Izuoka, Motoh Iwasa, Yoshiyuki Takei, Kenji Togashi

**Affiliations:** ^1^Center for Physical and Mental Health, Mie University Graduate School of Medicine, Tsu, Japan; ^2^Department of Gastroenterology and Hepatology, Mie University Graduate School of Medicine, Tsu, Japan; ^3^JST, PRETO, Kawaguchi, Japan; ^4^Department of Health Welfare Sciences, Kansai University of Welfare Sciences, Kashiwara, Japan; ^5^Department of Health and Physical Education, Mie University Faculty of Education, Tsu, Japan

**Keywords:** extracellular vesicle, proteomic analyses, antioxidant, short time of high intensity interval training exercise, skeletal muscle

## Abstract

**Introduction/Purpose:**

High-intensity interval training (HIIT) promotes various biological processes and metabolic effects in multiple organs, but the role of extracellular vesicles (EVs) released from a variety of cells is not fully understood during HIIT exercise (HIIT-Ex). We investigated the changes in circulating number and proteomic profile of EVs to assess the effect of HIIT-Ex.

**Methods:**

Seventeen young men (median age, 20 years) were enrolled in the study. Total duration of the HIIT-Ex was 4 min. Blood samples were collected from before HIIT-Ex (pre-HIIT-Ex), at the immediate conclusion of HIIT-Ex (T_0_), at 30 min (T_30_), and at 120 min after HIIT-Ex. The pulse rate and systolic blood pressure were measured. Circulating EVs were characterized, and EV proteins were detected *via* nano liquid chromatography tandem mass spectrometry.

**Results:**

The pulse rate and systolic blood pressure at T_0_ to pre-HIIT-Ex were significantly higher. Circulating EV number was significantly altered throughout the HIIT-Ex, and the source of circulating EVs included skeletal muscle, hepatocytes, and adipose tissue. Proteomic analysis identified a total of 558 proteins within isolated circulating EVs from pre-HIIT-Ex, T_0_, and T_30_. Twenty proteins in total were significantly changed at pre-HIIT-Ex, T_0_, and T_30_ and are involved in a variety of pathways, such as activation of coagulation cascades, cellular oxidant detoxification, and correction of acid–base imbalance. Catalase and peroxiredoxin II were increased at T_0_.

**Conclusion:**

The circulating EV composition can be immediately changed by particularly a short time of HIIT-Ex, indicating that EVs may intercommunicate across various organs rapidly in response to HIIT-Ex.

## Introduction

High-intensity interval training (HIIT) comprises repeated bouts of high-intensity exercise (e.g., running, cycling, or swimming) and recovery periods of either lower-intensity exercise or rest, which can be carried out using a variety of protocols that manipulate the duration of sprint and recovering interval, the intensity of exercise, and the number of repeated sets (Buchheit and Laursen, 2013a). HIIT promotes the processes of oxygen transport and utilization, which improves overall physical fitness by elevating maximal oxygen consumption (VO_2__max_) (Laursen and Jenkins, 2002). HIIT is known to improve the metabolic function of multiple organs including the heart, lungs, and skeletal muscle (Buchheit and Laursen, 2013a, b). HIIT, combined with caloric restriction, leads to increased glucose and lipid storage in skeletal muscle, as well as elevated adipose thermogenesis (Davis et al., 2017). HIIT has also been shown to improve insulin sensitivity among the elderly (Sogaard et al., 2018), indicating that HIIT promotes various alterations in biological processes and overall metabolism within multiple organs.

Extracellular vesicles (EVs) including exosomes as endosomal origins and microvesicles as blebbing from plasma membrane, which have been shown to be released from a variety of cells, are composed of a lipid bilayer embedded with membrane proteins and an aqueous core containing water-soluble proteins and nucleic acids (Tkach and Thery, 2016; Kalluri and LeBleu, 2020). EVs circulate within the blood; therefore, circulating EVs are useful as non-invasive biomarkers given their specific protein and nucleic acid composition. EVs indicate myriad pathological conditions from the presence of subclinical metabolic risk to the progression of an underlying disease and can predict the EV source: organs/tissues/cells (Kalluri and LeBleu, 2020). EVs are also a tool for cell-to-cell communication through the transfer of various proteins and genes, such as microRNAs and mitochondrial DNAs, to recipient cells that can then regulate a wide assortment of cellular processes (Kalluri and LeBleu, 2020). In general, EVs released from damaged/activated cells contribute to disease progression, while EVs released from embryonic and mesenchymal stem cells repair cellular damage and tissue injury (Kalluri and LeBleu, 2020). We have reported that circulating EV number was significantly associated with various metabolic parameters, including obesity and lipid and glucose metabolism in humans under healthy and aberrant metabolic conditions (Kobayashi et al., 2018). In addition, we found that circulating EV number was significantly associated with skeletal muscle volume, but only in healthy individuals, suggesting that skeletal muscle releases EVs to maintain normal physical response (Kobayashi et al., 2018). Indeed, exercise stimulates EV release from a variety of tissues including skeletal muscle and also changes EV composition (Fruhbeis et al., 2015; Whitham et al., 2018; Vechetti, 2019; Denham and Spencer, 2020). In addition, skeletal muscle releases various types of EVs (Rome et al., 2019), and C2C12 myoblast and myotube release exosome-like vesicles (Forterre et al., 2014) and microvesicles (Guescini et al., 2010; Highton et al., 2019), but the investigations into the impact of EVs on skeletal muscle are still insufficient, with only a handful of studies that investigated encapsulated microRNAs and a short list of proteins (Vechetti et al., 2020).

There have been several studies concerning EV release in human exercise models (Nederveen et al., 2020) such as 2-km run (Whitham et al., 2018) or 1 h bout of cycling Ex (Wu and Liu, 2018), although the profile of EVs is not fully identified during HIIT exercise (HIIT-Ex). In addition, prompt change of circulating EVs after HIIT-Ex with 4-min duration has not been examined.

In this study, we investigate the association between circulating EVs and a multiorgan response, including lipid and glucose metabolism during HIIT exercise (HIIT-Ex). We also provide data on the prompt change in circulating EV numbers and EV protein composition after HIIT-Ex with short duration.

## Materials and Methods

### Study Participants

Seventeen young male individuals (median age, 20 years) enrolled in the study. They were students of Mie University (Mie, Japan) majoring in health and physical education. They were physically active and were trained as players of the Mie University baseball team. The basic characteristics of the study participants are shown in Table 1. The study was approved by the Ethics Committee at Mie University (approval no. 3201). All study participants provided written informed consent prior to enrollment. All methods were performed in accordance with the relevant guidelines and regulations.

**TABLE 1:** Basic characteristics of subjects.

Parameter	Unit	Values (min–max)
N		17
Age	(y)	20 (20–21)
BMI	(kg/m^2^)	22.0 (19.6–26.6)
Lean body mass	(kg/m^2^)	19.4 (17.5–20.4)
SMV	(kg/m^2^)	18.4 (16.6–19.4)
Fat mass	(%)	14.4 (6.3–22.1)
Pulse rate	(beats/min)	72 (55–107)
Systolic BP	(mmHg)	123 (98–143)
Diastolic BP	(mmHg)	63 (50–80)
AST	(IU/L)	21 (13–25)
ALT	(IU/L)	16 (11–31)
g-GPT	(IU/L)	19 (12–44)
LDL-Chol	(mg/dl)	94 (42–135)
TG	(mg/dl)	67 (37–191)
Cr	(mg/dl)	0.85 (0.80–1.12)
FBG	(mg/dl)	83 (65–93)
Fasting IRI	(mU/ml)	4.09 (1.47–12.70)
HOMA-IR		0.84 (0.29–2.60)
HOMA-b		78.8 (31.1–394.2)

### HIIT-Ex Protocol and Blood Collection

Exercise was undertaken on a programmable bicycle ergometer (Powermax VII, Konami Corporation, Tokyo, Japan). Prior to the HIIT-Ex, VO_2__max_ value for each participant was obtained by gas exchange analysis during bicycle ergometer tests. A number of protocols exist for estimating VO_2__max_. In the present study, initial power output was set at 30 W, and the output was then gradually increased by 2 W every 4 s afterward up to all-out max effort. The pedal cycling speed was fixed at 60 revolutions per minute (rpm) throughout the analysis.

**FIGURE 1:**
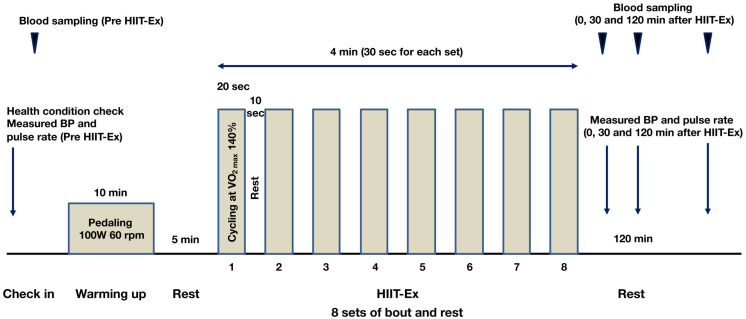
Protocol of high-intensity interval training exercise (HIIT-Ex). Eight sets, comprising exercise bout (cycling at 140% of VO_2__max_ for 20 s) and rest for 10 s, were performed during a single session of HIIT-Ex. W, watt; rpm, revolutions per minute.

The protocol of HIIT-Ex is shown in Figure 1. The protocol in the present study was modified from a protocol described by Tabata et al. (1996). Briefly, the warm-up was done by cycling at a rate of 60 rpm for 10 min. The power output during the warm-up was set at 100 W. After a 5-min rest, a single session of HIIT-Ex was done. Eight sets composed of an exercise bout (cycling at 140% of VO_2__max_ for 20 s) and rest for 10 s were performed during the HIIT session. Serum was separated from whole blood and was immediately frozen at −80°C until analysis.

### Collecting Anthropometric and Laboratory Data

We measured anthropometric data including body mass index (BMI), lean body mass, skeletal muscle volume (SMV), fat mass, pulse rate, systolic blood pressure (BP), and diastolic BP. Lean body mass, SMV, and fat mass were measured using multiple-frequency bioimpedance technology (MC-190 body composition analyzer, TANITA Corporation, Tokyo, Japan). Measurements of pulse rate, systolic BP, and diastolic BP, as well as blood collection were performed before HIIT-Ex (pre-HIIT-Ex), at the immediate conclusion of HIIT-Ex (T_0_), at 30 min (T_30_), and at 120 min (T_120_) after HIIT-Ex. Serum was separated from blood and immediately stored at −80°C for analysis.

Serum levels of aspartate aminotransferase (AST), alanine aminotransferase (ALT), γ-glutamyl transpeptidase (γ-GTP), low-density lipoprotein cholesterol (LDL-Chol), triglyceride (TG), creatinine (Cr), fasting blood glucose (FBG), free fatty acid (FFA), creatine phosphokinase (CPK), and fasting immunoreactive insulin (IRI) were measured at SRL Inc. (Tokyo, Japan). The homeostatic model assessment of insulin resistance (HOMA-IR) was calculated as [fasting IRI (μU/ml) × FBG (mg/dl)]/405 (Matthews et al., 1985). The homeostasis model assessment–β-cell function (HOMA-β) was calculated as [fasting IRI (μU/ml) × 360/FBG (mg/dl)] – 63 (Matthews et al., 1985).

### Flow Cytometry Analysis of Circulating EVs

Serum was centrifuged at 2,000 × *g* for 10 min to eliminate any aggregations. Circulating EVs in 2 μl of serum were stained with 4 μg/ml of calcein-AM (Invitrogen, San Diego, CA, United States) for 30 min in the dark at room temperature. The number of EVs was determined by flow cytometry (BD Cant II; BD Biosciences, San Jose, CA, United States) with flow cytometry alignment beads (Thermo Fisher Scientific, Tokyo, Japan) by triplication in each sample, and data were analyzed using FlowJo software (TreeStar, Ashland, OR, United States).

### Isolation of Circulating EVs and Measurement of Size

Serum was centrifuged at 2,000 × *g* for 10 min to eliminate any aggregations. Circulating EVs were isolated from an equal amount (500 μl) of serum between samples *via* qEV columns (Izon Science, Christchurch, New Zealand) according to the manufacturer’s instruction. All fractions, fractions 1–30, were collected with 500 μl each, and protein amount was measured at OD_280_ according to the manufacturer’s instruction. Fractions 6–10 were concentrated with Amicon Ultracel-3K (EMD Millipore, Temecula, CA, United States). The diameter of isolated circulating EVs was measured *via* nanoparticle tracking analysis, Nanosight LA10 (Malvern Panalytical, Malvern, United Kingdom) by triplication in each sample.

### Analysis of Protein Composition in Circulating EVs *via* Nano Liquid Chromatography Tandem Mass Spectrometry (Nano-LC-MS/MS)

The method for isolating EVs from serum was described above. We randomly picked up 3 subjects from the 11 subjects with elevated circulating EV numbers at T_0_ compared to pre-HIIT-Ex. Briefly, circulating EVs were isolated from an equal amount (500 μl) of plasma between samples *via* qEV columns (Izon Science) according to the manufacturer’s instruction. Fractions 6–10 were collected from the columns, and EVs were precipitated using trichloroacetic acid, followed by reduction, alkylation with iodoacetamide, and trypsinization using an equal amount of protein between samples. Samples were separated using nano-LC-MS/MS, EASY-nLC 1200 (Thermo Fisher Scientific), and Q Exactive Plus (Thermo Fisher Scientific) at APRO Science Institute (Tokushima, Japan). Data were analyzed using Scaffold4 (Proteome Software, Portland, OR, United States) against the SwissProt database at APRO Science Institute. Quantitative value (normalized total spectra) on Scaffold4 (Proteome software Inc., Portland, OR, United States) was used for heatmap and protein interactions, which were generated using Heatmapper software (Babicki et al., 2016) and STRING v11.0 software (Szklarczyk et al., 2019), respectively.

### Western Blotting Analysis of Isolated EVs

Isolated EVs (10.4 μg) were lysed with radioimmunoprecipitation assay (RIPA) lysis buffer resolved in TGX^TM^ precast gels and transferred to polyvinylidene difluoride membrane (BioRad, Hercules, CA, United States). Blotted membranes were incubated with blocking reagent for Can Get signal (TOYOBO, Osaka, Japan) and primary antibodies followed by peroxidase-conjugated secondary antibody (GE Healthcare Life Sciences, Pittsburgh, PA, United States) in Can Get solutions. The primary antibodies used were anti-CD9 (BioLegend, San Diego, CA, United States; 312102, ×500), anti-CD81 (SBI, Palo Alto, CA, United States; EXOAB-CD81A-1, ×1,000), anti-TSG101 (SBI; EXOAB-TSG101-1, ×1,000), anti-syntenin-1 (Santa Cruz, Dallas, TX, United States; 515538, ×1,000), anti-asialoglycoprotein receptor 1 (ASGPR1) (Genetex, Irvine, CA, United States; 122674, ×1,000), anti-perilipin (Abcam; 61682, ×1,000), anti-α-skeletal muscle actin (Abcam; 52218, × 500), anti-peroxiredoxin II (PRDX-2) (Santa Cruz; 515428, ×1,000), and anti-catalase (CAT) (Santa Cruz; 271803, ×1,000). The membrane was treated with Stripping buffer (Nakalai Tesque, Kyoto, Japan) or azide-TBST to remove horseradish peroxidase (HRP). Protein bands were visualized using enhanced chemiluminescence reagents (Thermo Fisher Scientific, Waltham, MA, United States) and digitized using a charge-coupled device camera (LAS4000 mini; Fuji Film, Tokyo, Japan). Expression intensity was quantified by Multi Gauge software (Fuji).

### Statistical Analysis

Data are expressed as medians with ranges. Parameter values determined at pre-HIIT-Ex, T_0_, T_30_, and T_120_ were compared using Kruskal–Wallis test. For proteomic data, parameter values in the entire three time points (at pre-HIIT-Ex, T_0_, and T_30_) were tested *via* mixed model repeated-measure analysis to determine the effect of time. Values with *p* < 0.05 were considered statistically significant. Statistical analysis was done using Multivariable Analysis for Mac version 3.0 software (Esumi, Tokyo, Japan) and SPSS version 22.0 J software (SPSS Japan, Tokyo, Japan).

## Results

### Basic Characteristics of All Participants

The basic characteristics of all subjects are summarized in Table 1. Values of liver enzymes, LDL-Chol, Cr, and FBG were within the normal range for all subjects. Two subjects had metabolic factor(s): one individual showed overweight (25.0 ≦ BMI < 30.0 kg/m^2^) (No authors listed, 2000), elevated serum TG ≧ 150 mg/dl, and elevated HOMA-R ≧ 2.5 (Matthews et al., 1985), and another individual showed elevated serum TG ≧ 150 mg/dl.

### Pulse Rate, Blood Pressure, Serum FFA Levels, and Circulating EV Number Were Changed by HIIT-Ex

**FIGURE 2:**
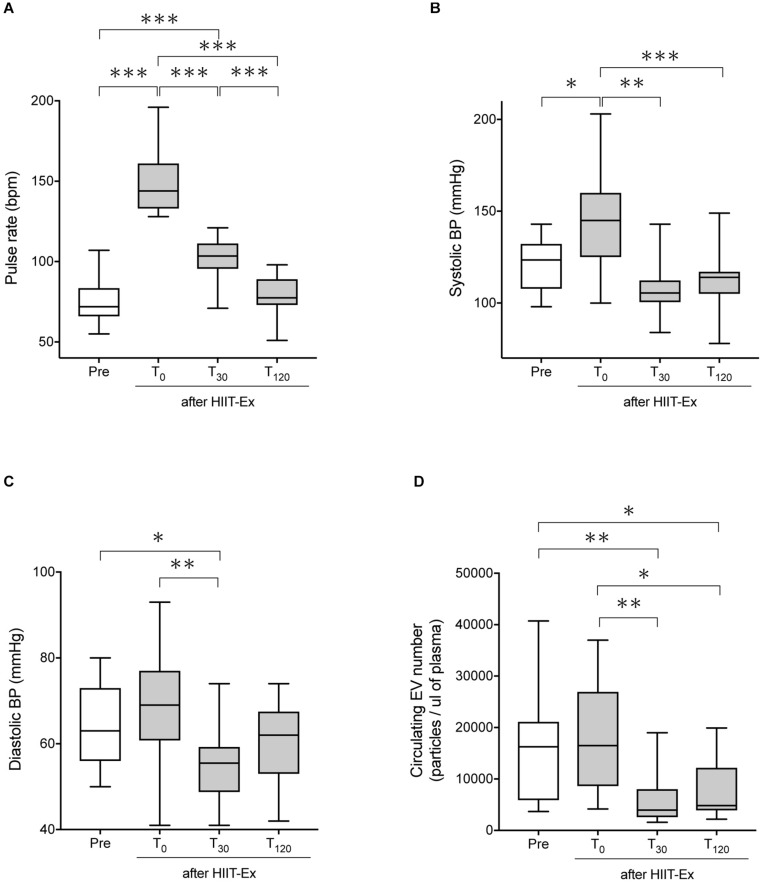
The pulse rate, systolic BP, diastolic BP, and circulating EV number before and after HIIT-Ex. Changes in pulse rate **(A)**, systolic blood pressure **(B)**, diastolic blood pressure **(C)**, and circulating EV number **(D)** in HIIT-Ex in all participants. Kruskal–Wallis test was used for statistical analysis. ^∗∗∗^*p* < 0.001. ^∗∗^*p* < 0.01, ^∗^*p* < 0.05. BP, blood pressure; bpm, beats per minute. Pre: pre-HIIT-Ex.

The protocol of HIIT-Ex is shown in Figure 1. Pulse rate and systolic BP were significantly elevated at T_0_ when compared to the values at pre-HIIT-Ex (pulse rate: *p* < 0.001, systolic BP: *p* < 0.05) (Figures 2A,B). Diastolic BP was not significantly increased at T_0_, whereas reduced at T_30_ (p < 0.05) when compared to the values at pre-HIIT-Ex (Figure 2C). Median values of systolic BP at T_30_ and T_120_ were lower than pre-HIIT-Ex, although the differences were not significant (Figure 2B). Serum levels of all other biochemical parameters including AST, CPK, Cr, LDL-Chol, and TG were not significantly changed after HIIT-Ex. Notably, serum FFA levels at T_120_ were significantly elevated compared to levels pre (*p* < 0.05), at T_0_ (*p* < 0.01), and at T_30_ (*p* < 0.01) (Supplementary Table 1). Next, we investigated the changes in circulating EV number as a result of HIIT-Ex using calcein AM, which was non-fluorescent until it passively enters EVs, after which it is activated by esterase and becomes fluorescent and EV impermeant (Gray et al., 2015; Figure 2D). The median value for circulating EV number in all subjects at pre-HIIT-Ex was 16,253 particles/μl, with a range of 3,654–40,725 particles/μl. EV number was significantly reduced at T_30_ and T_120_ when compared to pre-HIIT-Ex (*p* < 0.01: T_30_ vs. pre-HIIT-Ex, *p* < 0.05: T_120_ vs. pre-HIIT-Ex). EV numbers at T_0_ was not significantly different from the values at pre-HIIT-Ex, although we found that circulating EV number was promptly increased in 11 out of 17 (64.7%) subjects at T_0_ when compared to pre-HIIT-Ex.

### Circulating EVs Were Derived From Skeletal Muscle, Hepatocytes, and Adipose Tissue

**FIGURE 3:**
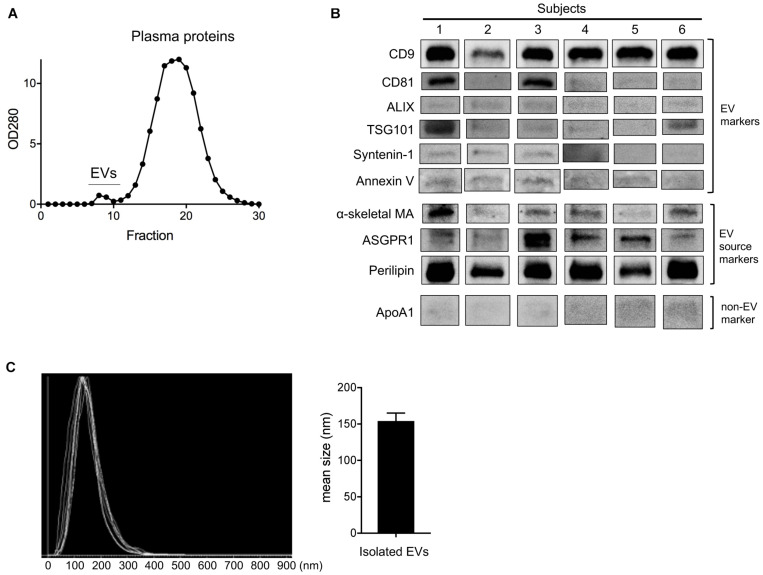
Purification and detection of EV source from circulating EV at T_0_. **(A)** Purification of circulating EVs using qEV column. **(B)** Representative immunoblotting analysis of CD9, CD81, ALIX, TSG101, syntenin-1, annexin V, α-skeletal muscle actin (α-skeletal MA), asialoglycoprotein receptor 1 (ASGPR1), perilipin, and apolipoprotein A1 (ApoA1) in isolated EVs from six subjects. **(C)** EV size of isolated EVs from six subjects by nano tracking analysis (NTA). Bar graph of isolated EV size from NTA analysis.

Changes in circulating EV number as a result of HIIT-Ex led us to further investigate the tissue and/or cellular source of EV release, as well as the overall EV protein composition. Therefore, we isolated circulating EVs from serum using qEV columns (Figure 3A) and detected exercise-induced metabolic proteins related to skeletal muscle, adipose, and liver in representative six subjects. We detected CD9, CD81, ALIX, TSG101, syntenin-1, annexin V (EV markers), α-skeletal muscle actin (α-skeletal MA—enriched in skeletal muscle) (Ilkovski et al., 2005), ASGPR1 (enriched in hepatocytes) (Ashwell and Morell, 1974; Povero et al., 2014), and perilipin A (enriched mainly in the adipose tissue) (Kimmel et al., 2010; Eguchi et al., 2016) in circulating EVs taken at T_0_ (Figure 3B and Supplementary Figure 1), suggesting that a part of circulating EVs were derived from multiple tissues and cells including the skeletal muscle, hepatocytes, and adipose tissue as a function of HIIT-Ex. Although we used qEV column that has less lipoprotein overlap for EV isolation, a small amount of apolipoprotein A1 was still detected as a contamination in all circulating EVs (Figure 3B and Supplementary Figure 1). The diameters of circulating EVs from six subjects were similar with average mean of 154.2 nm (Figure 3C).

### HIIT-Ex Induced Changes in Circulating EV Protein Composition Involving Cellular Oxidant Detoxification, Regulation of Exocytosis, Regulation of Stress, Regulation of Vesicle-Mediated Transport, Extracellular Matrix Organization, and Response to Stimulus

**FIGURE 4:**
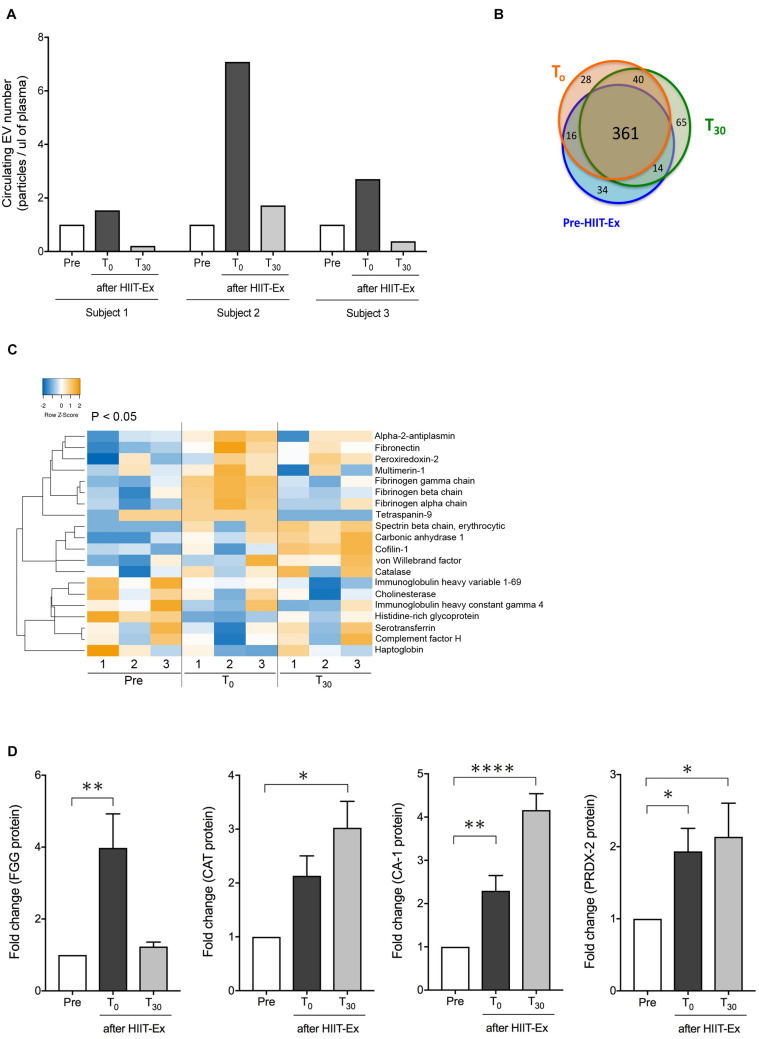
Detected EV proteins. **(A)** Changes in circulating EV number at pre-HIIT-Ex, T_0_, and T_30_ in three subjects using proteomic analysis. **(B)** Detected protein number at pre-HIIT-EX, T_0_, and T_30_. **(C)** Heatmap of identified proteins with significant changes in isolated circulating EVs at pre-HIIT-Ex, T_0_, and T_30_. **(D)** Change in EV protein values at pre-HIIT-Ex, T_0_, and T_30_. Fibrinogen gamma chain (FGG), catalase (CAT), carbonic anhydrase (CA-1), and peroxiredoxin II (PRDX-II). Kruskal-Wallis test was used for evaluating statistical significance among the three sampling points. ^****^p < 0.0001, ^∗∗^*p* < 0.01, ^∗^*p* < 0.05. Pre: pre-HIIT-Ex.

Next, we explored circulating EV composition in three individuals using proteomic analysis. Three individuals were randomly picked from the 11 participants with elevated circulating EV numbers at T_0_ compared to pre-HIIT-Ex. The circulating EVs from each participant were elevated at T_0_ when compared to pre-HIIT-Ex and were decreased at T_30_ (Figure 4A). These results were ascertained in terms of the EV proteome by nano-LC-MS/MS analysis. The proteomic analysis identified a total of 558 proteins contained within isolated circulating EVs from pre-HIIT-Ex, T_0_, and T_30_. Of the 558 proteins, a total of 361 proteins were found to be common among circulating EVs taken from all time points. Drilling down further within this protein pool, we determined a total of 34, 28, and 65 proteins unique to pre-HIIT-Ex, T_0_, and T_30_, respectively (Figure 4B). We observed significant changes in a total of 20 proteins including alpha-2-antiplasmin, von Willebrand factor (VWF), multimerin-1 (MMR1), fibrinogen alpha chain (FGA), fibrinogen beta chain (FGB), fibrinogen gamma chain (FGG), carbonic anhydrase-1 (CA-1), PRDX-2, catalase (CAT) the above-mentioned CAT, and CA-1 within circulating EVs among samples taken at pre-HIIT-Ex, T_0_, and T_30_ (Figure 4C). FGG, an essential component to form an insoluble fibrin matrix in hemostasis, was increased significantly at T_0_ compared to pre HIIT-Ex (*p* < 0.01) (Figure 4D). An antioxidative protein CAT was gradually increased to be significantly elevated at T_30_ compared to pre-HIIT-Ex (*p* < 0.05) (Figure 4D). CA-1, as an essential protein for maintaining acid–base homeostasis, was significantly increased both at T_0_ and T_30_ compared to pre-HIIT-Ex (*p* < 0.01 T_0_ vs. Pre-HIIT-Ex, p < 0.0001 T_30_ vs. pre-HIIT-Ex) (Figure 4D). An antioxidant protein PRDX-2 was significantly increased both at T_0_ and T_30_ compared to pre-HIIT-Ex (*p* < 0.05 T_0_ vs. pre-HIIT-Ex, p < 0.05 T_30_ vs. pre-HIIT-Ex) (Figure 4D). We listed 12 proteins that were significantly increased at the immediate conclusion of HIIT-Ex (T_0_) (each *p* < 0.05) (Table 2).

**TABLE 2:** EV proteins significantly elevated at T_0_.

Protein ID	*p*-value (Pre. vs. T_0_)
Alpha-2-antiplasmin	0.011*
Fibronectin	0.011*
Peroxiredoxin-2	0.021*
Fibrinogen gamma chain	0.009**
Fibrinogen beta chain	0.029*
Fibrinogen alpha chain	0.021*
Carbonic anhydrase 1	0.004**
Immunoglobulin heavy constant gamma 4	0.021*
Histidine-rich glycoprotein	0.003**
Serotransferrin	0.019*
Haptoglobin	0.018*
Reelin	0.049*

*p < 0.05, **p < 0.01, analyzed by Kruskal–Wallis test.

**FIGURE 5:**
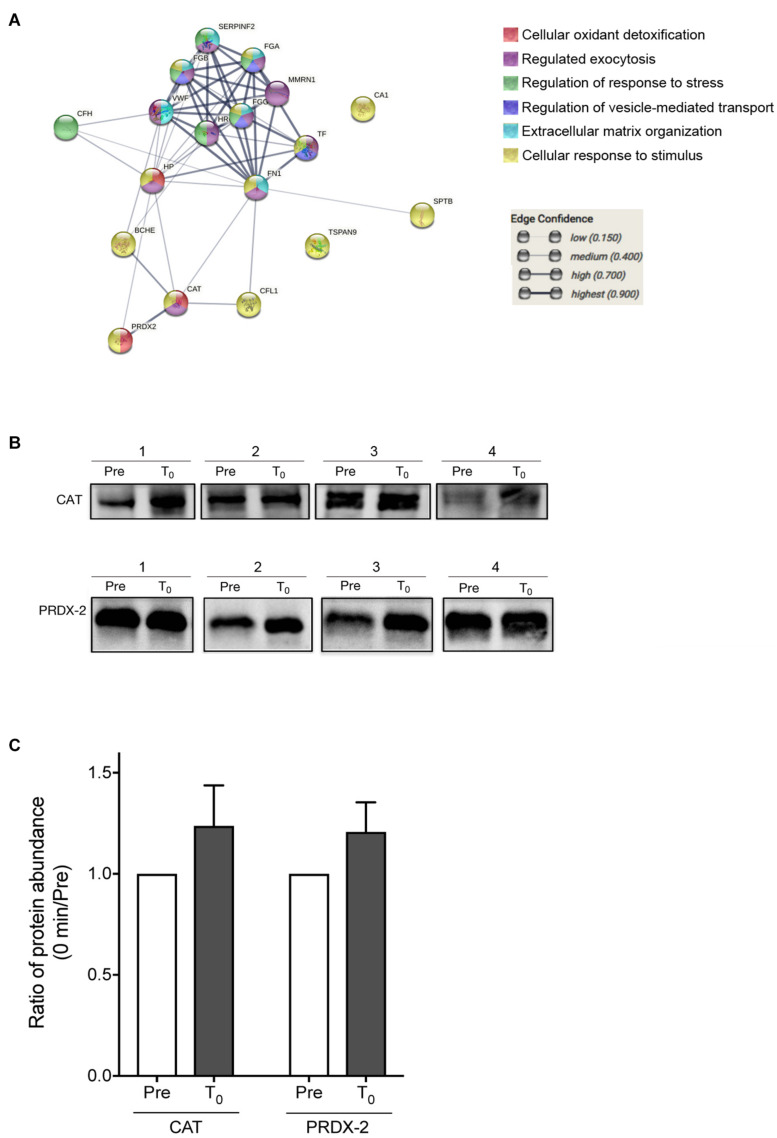
Proteomics analysis of EV proteins. **(A)** Proteomics analysis was performed on the EV proteins changed among at pre-HIIT-Ex, T_0_, and T_30_. **(B)** Representative immunoblotting of CAT and PRDX-2 in circulating EVs before (pre) and at T_0_. **(C)** Abundance ratio of EV protein expression, CAT and PRDX-2, in circulating EVs at T_0_ compared to pre-HIIT-EX, which was quantified using Multi Gauge software. Pre: pre-HIIT-Ex.

From the protein–protein interaction analysis using STRINGS, the 20 proteins, which were significantly changed during HIIT-Ex, elucidated various protein–protein interaction clusters including cellular oxidant detoxification, regulation of exocytosis, regulation of stress, regulation of vesicle-mediated transport, extracellular matrix organization, and response to stimulus (Figure 5A and Supplementary Table 2). Notably, we found that antioxidant enzymes, CAT and PRDX-2, were increased at T_0_ and T_30_ (Figure 4D), leading us to further validate the abundance of CAT and PRDX-2 in circulating EVs using Western blotting. We detected abundance of CAT and PRDX-2 at pre-HIIT-Ex and T_0_ in representative four subjects (Figure 5B and Supplementary Figure 2). The abundance ratio of antioxidant proteins CAT and PRDX-2 in the isolated circulating EVs of all subjects was increased at T_0_ (Figure 5C).

## Discussion

In the current study, we investigated the changes in circulating number and proteomic profile of EVs to assess the effect of HIIT-Ex. We have demonstrated that the circulating EV number is significantly changed at 30 or 120 min after HIIT-Ex, and overall EV protein composition including FGG, CAT, CA-1, and PRDX-2 is significantly increased at 0 or 30 min after HIIT-Ex. We also showed that a part of circulating EVs were derived from multiple organs including skeletal muscle, adipose tissue, and the liver, suggesting possible interorgan network by HIIT-Ex. We have previously reported a strong correlation between the level of circulating EVs and lipid metabolism, especially with respect to serum TG levels (Kobayashi et al., 2018). Indeed, in the present study, circulating EV number was reduced at T_30_ in concordance with the reduction in serum levels of TG and FFA. Notably, serum FFA levels were significantly increased at T_120_, suggesting that HIIT-Ex can facilitate FFA release from the organs, including white adipose tissue, to compensate for overall energy loss as a result of HIIT-Ex.

The number of circulating EVs was elevated just after HIIT-Ex in 11 out of 17 subjects (64.7%). It was surprising that EVs were promptly released into the systemic circulatory pathway in response to only 4 min of exercise intervention. Although we did not observe significant elevation of circulating EV number in all subjects, this result would be expected because HIIT-Ex was performed only for 4 min and circulating EV number at pre-HIIT-Ex was influenced by healthier condition, such as TG levels and inflammation. Furthermore, the efficacy of cardiopulmonary and skeletal muscle loads was different in each subject. Circulating EV number was significantly reduced at T_30_ in all subjects, suggesting that EVs can be received and processed by their target cells, or simply degraded.

Notably, EV proteins were dramatically changed by HIIT-Ex. EV contains a variety of protein types involved in platelet aggregation and coagulation cascades (MMRN1, VWF, FGA, FGB, FGG, and alpha-2-antiplasmin), acid–base homeostasis (CA-1), and antioxidant (CAT and PRDX-2). FGG, which is involved in coagulation cascades including fibrinolysis, was elevated at T_0_. HIIT-Ex is known to promote changes in hemostatic and fibrinolytic properties. Short-duration HIIT-Ex significantly increased plasma concentrations of tissue factor, tissue factor pathway inhibitor, thrombin–anti-thrombin complex, and D-dimer (Zadow et al., 2018), resulting in the changes in hemostatic and fibrinolytic properties. The significant increase in EV proteins involved in coagulation cascades found in the present study suggests the activation of the coagulation pathway just after HIIT-Ex, which is in line with previous studies. CA-1 in EVs was also increased after HIIT-Ex. CA-1, one of the cytosolic carbonic anhydrase isozymes, catalyzes the interconversion between [CO_2_ + H_2_O] and [H^+^ + HCO_3_^–^], which is an essential reaction for maintaining acid–base homeostasis in multiple organs. Intensive muscular exercise induces the formation of lactate and CO_2_, which leads to intracellular acidosis. A previous study reported increased blood CA activity 2 weeks after starting interval training (Tas et al., 2019). Therefore, the prompt elevation of CA-1 in EVs may be an adaptive reaction to correct for an acid–base imbalance brought on by HIIT-Ex.

Muscular Ex is associated with an increase in overall oxidative stress (Dillard et al., 1978). Intracellular radicals comprise reactive oxygen species and reactive nitrogen species. Many studies have demonstrated that short-duration and high-intensity Ex promoted an increase in oxidative stress in both blood and skeletal muscle (Powers et al., 2016) due to the contraction of skeletal muscle producing free radicals (Reid et al., 1992). Intracellular radicals play an adverse role in regulating muscle fatigue and the production of skeletal muscle force (Powers and Jackson, 2008). Therefore, antioxidant mechanisms are required to ameliorate muscle cell damage. Human CAT predominantly catalyzes the dismutation of hydrogen peroxide into oxygen and water (H_2_O). Our results were consistent with the previous report in which it was demonstrated that plasma CAT levels were significantly increased at 3 and 24 h after HIIT-Ex (Bessa et al., 2016). PRDX-2, a member of the PRDX family, is abundant in erythrocytes, skeletal muscle, and cardiac muscle (Brinkmann and Brixius, 2013). Moghaddam et al. (2011) reported that PRDX-2 in erythrocytes was upregulated *via* physical training (3 months, three times a week, cycling for 25–50 min at 75% of maximal heart rate) in patients with type 2 diabetes. Conversely, another study reported that there was no change in skeletal muscle PRDX-2 expression in patients with type 2 diabetes after training (3 months, twice a week, cycling for 25–50 min at heart rate corresponding to 2 n mon/L blood lactate concentration) (Brinkmann et al., 2012). The current study revealed that these antioxidant enzymes were encapsulated in EVs of circulating blood, although we need to investigate the regulation manner of the encapsulated PRDX-2 driven by HIIT-Ex in future studies.

CA and PRDX-2 have been identified as significant elevated EV-packed proteins in different types of Ex, such as 2-km run (Wu and Liu, 2018) and 1 h bout of cycling Ex (Whitham et al., 2018). We demonstrated these proteins encapsulated in EVs were promptly released into circulation in response to only 4 min of HIIT-Ex, suggesting they can be used as biomarkers for HIIT-Ex with a particularly short time, as well as other type of Ex, although we needed further validation in larger cohorts.

The present study has some limitations. (1) The current study was a prospective single-arm study, not a randomized study with a control group. (2) The sample number of proteomics analysis was limited due to expensive cost. In the current study, proteomics analysis at pre-HIIT-Ex, T_0_, and T_30_ was performed on three subjects. We detected catalase and PRX2 in the remaining 14 subjects by Western blotting. In the future, we need to validate the current results with proteomics analysis in a larger number of samples.

## Conclusion

**FIGURE 6:**
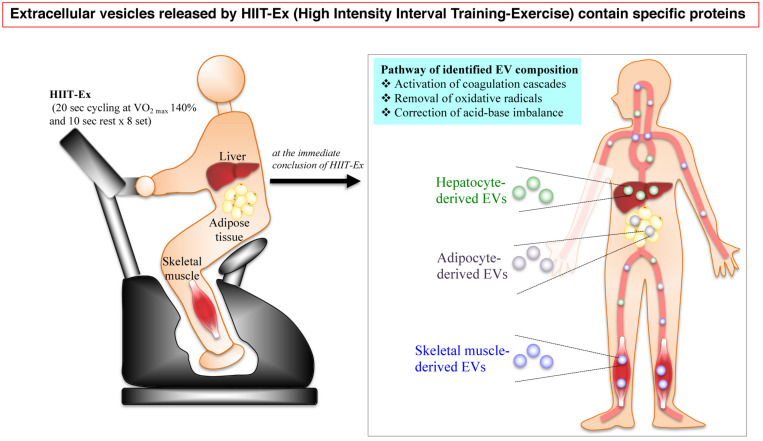
A schematic illustration of the HIIT-EX advantage on EV release from organs associated with metabolism. HIIT-EX enhances EV release from organs including liver, adipose tissue, and skeletal muscle associated with metabolism. These EVs contain specific proteins relating to several pathways.

In conclusion, the circulating EV numbers and protein composition can be immediately changed after 0 and/or 30 min by a particularly short time of HIIT-Ex. A part of circulating EVs were derived from multiple tissues and cells including the skeletal muscle, hepatocytes, and adipose tissue as a function of HIIT-Ex. Furthermore, the protein composition of EVs involved in activating coagulation cascades, removal of oxidative stress, and correction of acid–base imbalance was identified by proteomic analysis. These results indicated that EVs intercommunicate across various organs rapidly in response to HIIT-Ex (Figure 6).

## Data Availability Statement

The datasets presented in this study can be found in online repositories. The names of the repository/repositories and accession number(s) can be found below: https://doi.org/10.6084/m9.figshare.13480317.v1.

## Ethics Statement

The studies involving human participants were reviewed and approved by the Ethics Committee at Mie University. The patients/participants provided their written informed consent to participate in this study.

## Author Contributions

AE, YK, and KT contributed to conception and design of the study. YK, AE, MI, and YTak organized the database. YTam, MT, and KI performed the experiments. SF performed the statistical analysis. YK wrote the first draft of the manuscript. AE and SF wrote sections of the manuscript. All authors contributed to manuscript revision, read, and approved the submitted version.

## Conflict of Interest

The authors declare that the research was conducted in the absence of any commercial or financial relationships that could be construed as a potential conflict of interest.

## Supplementary Material

Supplementary Figure 1 The whole membrane of Western blotting for Figure 3B.

Supplementary Figure 2 The whole membrane of Western blotting for Figure 5E.

Supplementary Table 1 Biochemical parameters before and after HIIT-Ex.

Supplementary Table 2 The list of proteins.
