# Supplementary figures and images for: Protein Composition of Circulating Extracellular Vesicles Immediately Changed by Particular Short Time of High-Intensity Interval Training Exercise
Source: Front Physiol. 2021 Jul 1;12:693007. doi: 10.3389/fphys.2021.693007 (PMC8280769; doi:10.3389/fphys.2021.693007)

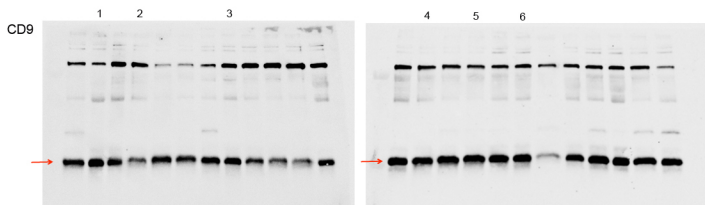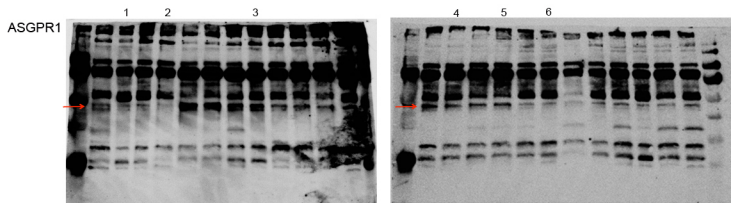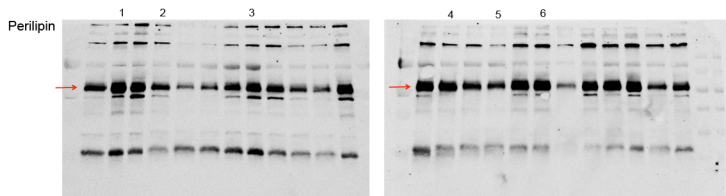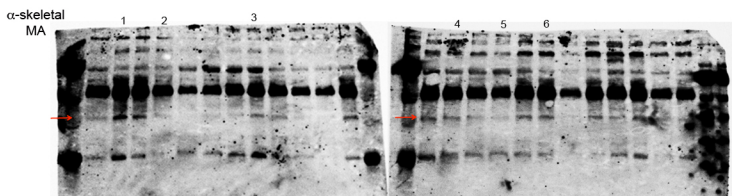

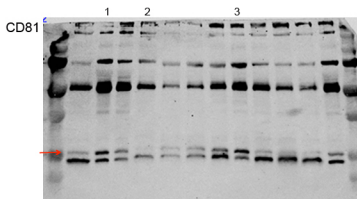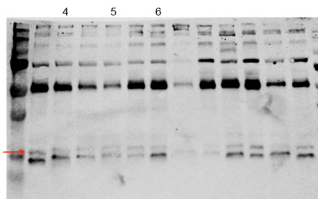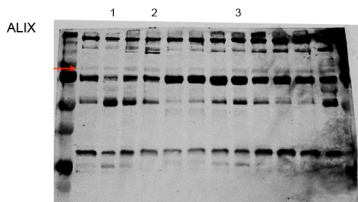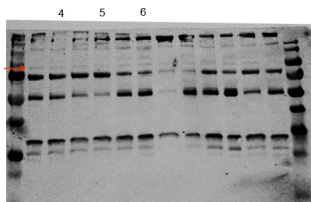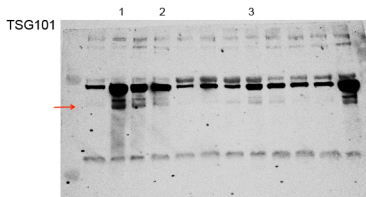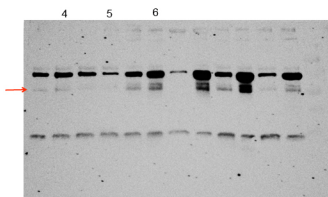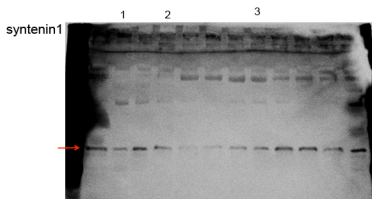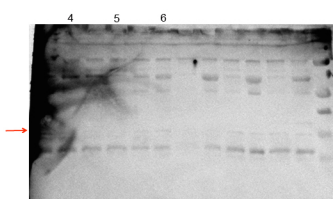

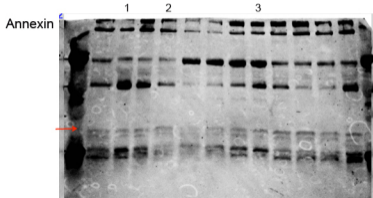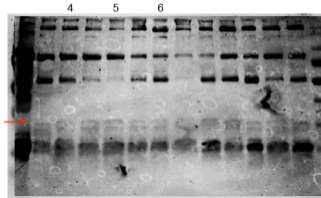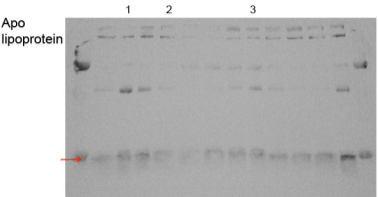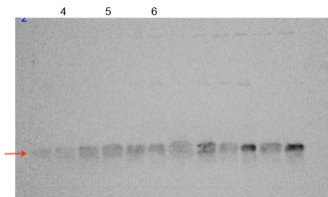

Supplement: Supplementary Figure 1 — The whole membrane of Western blotting for Figure 3B. [file Data_Sheet_1.PDF]

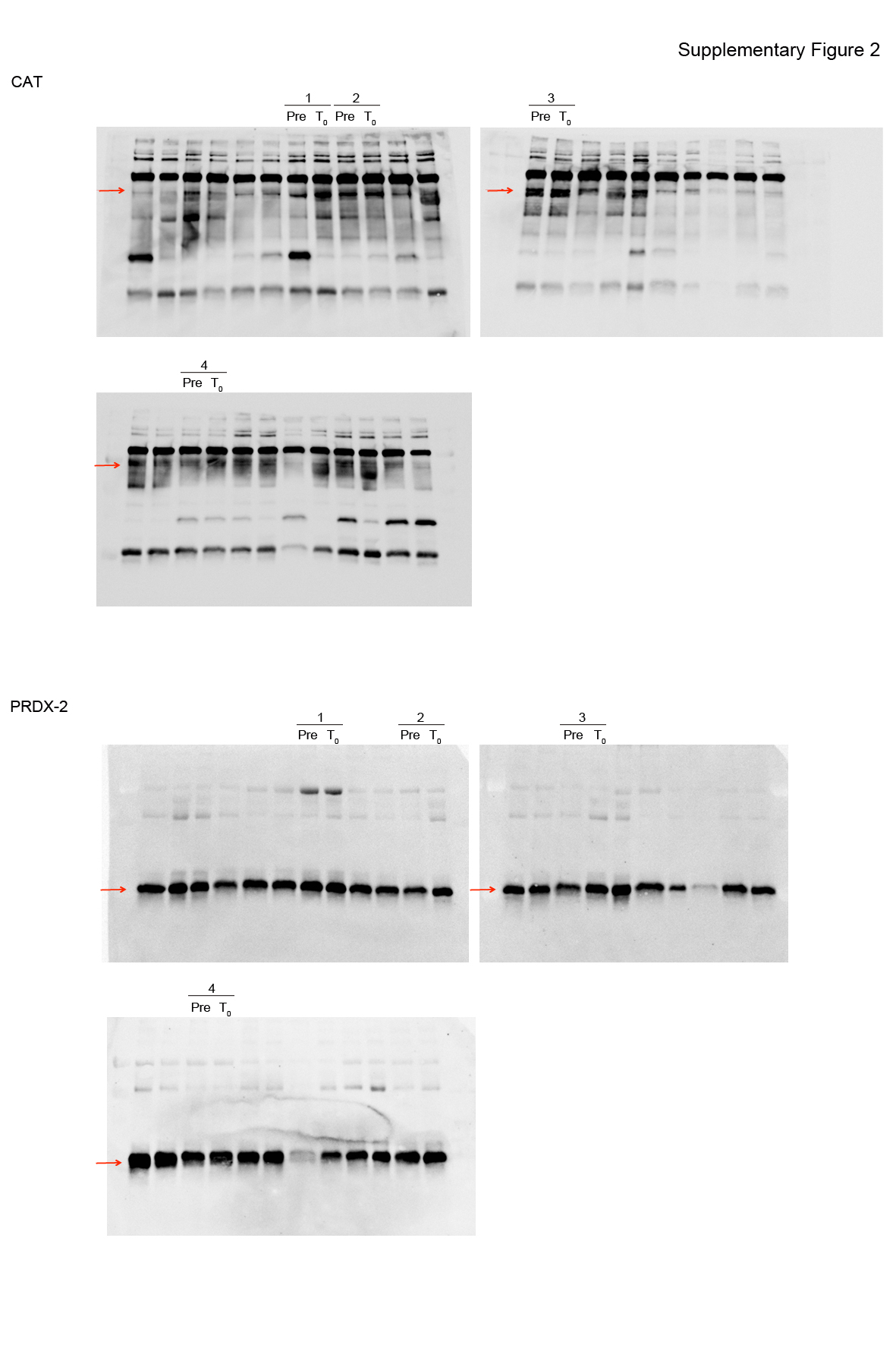

Supplement: Supplementary Figure 2 — The whole membrane of Western blotting for Figure 5E. [file Image_1.JPEG]
