# Supplementary material for: Protein Composition of Circulating Extracellular Vesicles Immediately Changed by Particular Short Time of High-Intensity Interval Training Exercise
Source: Front Physiol. 2021 Jul 1;12:693007. doi: 10.3389/fphys.2021.693007 (PMC8280769; doi:10.3389/fphys.2021.693007)
Supplement: Supplementary Table 1 — Biochemical parameters before and after HIIT-Ex. [file Table_1.DOCX]

**Supple. Table 1. Biochemical parameters before and after HIIT-Ex**

| Parameter | Pre | After HIIT-Ex | | |
| --- | --- | --- | --- | --- |
|  |  | **T_0_** | **T_30_** | **T_120_** |
| AST (IU/L) | **21 (13-37)** | **24 (16-30)** | **23 (16-39)** | **22 (15-37)** |
| CPK (IU/L) | **190 (94-576)** | **228 (108-662)** | **209 (109-598)** | **196 (101-549)** |
| Cr (mg/dL) | **0.85 (0.80-1.12)** | **0.97 (0.88-1.16)** | **0.91 (0.81-1.10)** | **0.91 (0.81-1.10)** |
| LDL-Chol (mg/DL) | **94 (42-135)** | **105 (48-148)** | **94 (43-141)** | **95 (42-132)** |
| TG (mg/dL) | **67 (37-191)** | **70 (37-193)** | **52 (30-174)** | **57 (35-154)** |
| FFA (mg/dL) | **514 (201-972)** | **416 (183-977)** | **330 (156-648)** | **836(436-1398) *** |

*p <0.05 (vs. pre) and p< 0.01 (vs. T_0_ and vs. T_30_), analyzed by Kruskal-Wallis test.
